# American Academy of Dental Science

**Published:** 1894-07

**Authors:** 


### AMERICAN ACADEMY OF DENTAL SCIENCE.

THE regular monthly meeting of the American Academy of Dental Science was held at Young's Hotel, Boston, on Wednesday evening, March 7, at six o'clock, President Smith in the chair. Papers for the evening were read as follows:

Charles F. Stacey, M.D.: subject, "Ocular Headaches." Mr. H. Carlton Smith: subject, "A Simple Test for Cocaine in Local Anæsthetics."

(For Dr. Stacey's paper, see page 431.)

### DISCUSSION.

*Dr. Hitchcock.*—I would like to ask Dr. Stacey what is the greatest variation in the lens?

*Dr. Stacey.*—The variation in the lens as regards sight is generally quite small. Where there is considerable variation, it is usually present both in the lens and the cornea. If large errors exist, oftentimes the lens is absent and the trouble is congenital, the patient being born without any lens at all. Then, of course, you have to replace the lens by a glass in front of the eye. That is what happens in the operation known as the "removal of a cataract," which means, in fact, the removal of the lens; and then you supply a lens by a strong glass in front of the eye.

*Dr. Hitchcock.*—I meant more especially the linear measure of the variation. Individuals differ in the convexity of the lens. In taking a relative linear measure, what is the greatest difference?

*Dr. Stacey.*—I cannot give you just that fact.

*Dr. Hitchcock.*—Suppose there are two people having lenses of a given convexity, would the sight be the same in both persons?

*Dr. Stacey.*—Oh, yes. Take the normal eye, for instance: the power of sight is always the same. A great many observers claim that there is no normal eye; that there is a certain amount of astigmatism in every individual; but cases have been reported where normal eyes were present, and I think I have seen one or two instances of it myself, although it is a rare thing. Where the curvature and convexity are the same, the sight should be the same.

*Dr. Hitchcock.*—I was thinking that what might be normal in one individual would be abnormal in another.

*Dr. Stacey.*—That is possible, but I should say not very probable.

*Dr. Hitchcock.*—I know that with my own eyes one of them is far-sighted and the other is supposed to be about normal, and the far-sighted eye was somewhat strained in trying to keep up to the normal eye, and yet the linear measure of the lens is very nearly the same.

*Dr. Stacey.*—That is very likely.

*Dr. H. A. Baker.*—I would like to ask Dr. Stacey if in case a person has astigmatism, and that is corrected by glasses and ocular headaches prevented, whether that fact proves that you have the correct glasses?

*Dr. Stacey.*—No; I don't think it does. Oftentimes you will put a glass onto a patient, and it will relieve the headache. In a year or more that patient returns, complaining of some trouble of the eyes, and you examine them and find that the glass is not just what it should be; and as a year's time should not make much difference in the power of the eye, it proves that you did not get the correct error when you first examined. You know in the examination of the eye we are obliged to effect a paralysis by putting in atropia, and there are some people who object to that; they won't let you put anything in their eyes at all, and consequently you are obliged to fit the glasses without atropinization. In this way you do not get the full error, and when the person returns the only thing to do is to atropinize the eye to get at the full error. Those persons may not suffer with headache, because a partial correction of the error will oftentimes relieve the headache; but they may have some other trouble which is a distinct symptom of impaired sight.

*President Smith.*—Is there any danger in using atropia in the eye?

*Dr. Stacey.*—There is very little danger in using such solutions of atropia as are ordinarily used. The strength of the solution most often used is two per cent., but in severe cases we sometimes use a four-per-cent. solution, and that is as strong as is ever put in the eye. The drug mostly used nowadays is called "homatropine," which is a somewhat modified preparation of atropine, and with that the paralysis generally lasts only twenty-four to forty-eight hours. I never heard of but one or two cases where it exceeded that length of time. In one case the paralysis lasted for four days, but the patient was a very nervous person, and it was thought that this result was owing to the neurasthenic condition of the patient.

*Dr. Eames.*—I would like a little fuller explanation in regard to the character of ocular headaches. Are they continuous headaches, or are they likely to be periodical, or brought on by some indisposition ?

*Dr. Stacey.*—The headaches may be periodical, or they may be constant; and when the error in the eyes is far-sighted astigmatism the trouble is increased when the eyes are focussed for near work; whereas, in near-sighted astigmatism the pain is increased by focussing the eyes at a distance. In the latter cases headaches are brought on by attending the theatre or a concert, or going out walking and taking vision at a parallel range; while oftentimes for near work the person is not affected at all, and gets along very comfortably with ordinary duties.

*Dr. Eames.*—I suppose that after a night's rest, and the eyes being refreshed, the patient would get relief from the headache, and the unpleasant symptoms would return with the use of the eyes?

*Dr. Stacey.*—Rest might relieve them to a certain extent, but the headache would return with the use of the eyes.

*Dr. Bradley.*—Some one asked regarding the effects of an indisposition. I would like to ask whether indigestion or a derangement of the functions of the stomach would affect the eyes?

*Dr. Stacey.*—As a rule, anything like that would tend to aggravate the trouble.

*Dr. Paine.*—Will Dr. Stacey please tell us if a trouble of the eye would cause indigestion?

*Dr. Stacey.*—Well, that opens up another factor. Some men have gone so far as to claim that almost all neurasthenic diseases can be traced to the eye. Dr. Stevens, of New York, who is a great eye-man, seems to hold this opinion. Epilepsy has been proved to have been caused by straining the eyes; nausea and vomiting have been produced by eye-strain; and it is a well-believed fact now that in a great many cases St. Vitus's Dance, or Chorea, is caused by eye-strain. Headaches, neurasthenic diseases, and in some cases stomach trouble, such as vomiting and vertigo, are what you might call occasional symptoms of an error in the refraction of the eyes.

*Dr. G. S. Baker.*—I would like to ask whether an abnormal condition of the eye is more common in females than in males?

*Dr. Stacey.*—I never happened to see any statistics in that respect, but I should say from what I have seen and what I have read that the abnormal condition is nearly equally distributed among the male and female.

*President Smith.*—If there are no further remarks to be made on this subject, we will pass to the next subject of the evening. I have the pleasure of introducing to you Mr. H. Carlton Smith. Mr. Smith will read a paper entitled "A Simple Test for Cocaine in Local Anæsthetics."

(For Mr. Smith's paper, see page 437.)

DISCUSSION.

*Dr. Hitchcock.*—I would like to ask Mr. Smith if he is entirely opposed to cocaine?

*Mr. Smith.*—No; I don't mean to give that impression. It may be a very valuable remedy to use if you know the strength of the solution you are using. I should oppose the use of cocaine in preparations that you know nothing whatever about. For instance, in one of the solutions that I examined, as I have told you here this evening, I found nearly four per cent. of cocaine. I should think it unwise to use such a preparation.

*Dr. Hitchcock.*—In speaking of "Dorsenia," you said you found one-fifth of one per cent., which is one in five hundred. There are four hundred and eighty minims in an ounce. If you use twenty minims for an injection, is not that perfectly safe?

*Mr. Smith.*—So far as the cocaine goes, that would be perfectly safe. In talking with one of your members before supper, he told me of a case where serious results followed the use of "Dorsenia." I should not expect serious effects from the cocaine there is in it, as it is such a slight amount. What else it may contain which would produce such results I do not know, as I simply tested for cocaine.

*Dr. Cooke.*—This "Dorsenia" which Mr. Smith had to-night is some which the proprietor and discoverer of the remedy gave me two or three years ago. I never used it, but kept it in my cabinet, and when I spoke to Mr. Smith, some time ago, about this matter, I handed it to him, and he analyzed it with the result which he has told you to-night. "Torpidus" Hood & Reynolds sell, and I was assured that there was only a small percentage of cocaine in it,—not enough to do any harm. "Muraline" B. L. Knapp & Co. keep, and I believe it is simply used to put in the pockets in pyorrhœa.

This is a subject that I am glad to have opened, as I never dared inject any of these substances under the gum. One year ago I decided to put aside everything that came to me in the shape of circulars or advertisements of anæsthetics; and I looked into the drawer the other day and found I had about twenty-five or thirty, a large share of the substances being used to inject under the gum for the extraction of teeth without pain; and as all these preparations have come since the discovery of cocaine, I suspect that cocaine is at the bottom of the whole of them.

*Dr. Potter.*—I am very glad that we are getting onto this line of work; it is just what we need to do. I cannot conceive how any one in our profession can use a secret remedy. If anything unfavorable should happen and the practitioner were brought up before the law, it would not help him at all to state that a certain manufacturing company said that the preparation used was entirely harmless. The practitioner must himself know the exact nature of the drug and its strength. There are too many in our profession who are using whatever the manufacturing chemists send to them. I think cocaine is a very valuable remedy, but when we use it we ought to know just what amount we have got under our thumb in the syringe, and so use a medicinal dose and not a dangerous one.

*Dr. Briggs.*—I would like to follow right on here and add that it seems to me the danger is not so much in the dentist's use of cocaine as in using one of these nostrums and supposing that he is not using cocaine, and in many cases probably entirely unprepared to treat the case should he have untoward results. There is where the greatest danger lies. Of the men who use those nostrums there is perhaps not one in one hundred who would know what to do in case of cocaine-poisoning, or has the means at hand to resuscitate the patient. Of course, where the doses run up high the danger is increased; but still, if a patient has an idiosyncrasy which prohibits the use of cocaine or other alkaloids, I have found that the smallest dose may give rise to dangerous symptoms; and the question is, "Is the dentist prepared to treat patients for cocaine-poisoning?"

*President Smith.*—Perhaps it would be well for Dr. Briggs to state what preparation is needed.

*Dr. Briggs.*—That is entering on another subject. I make it a point to always have on hand the heart-stimulants, the usual restoratives for heart-failure, with the particular addition of always keeping capsules of nitrite of amyl, which seems to be specially useful in cases of cocaine-poisoning. In administering the drug, whenever I have seen any signs of absorption I have given ammonia by inhalation and by the stomach, and in one or two cases subcutaneous injections of brandy or ether. These are standard remedies which every dentist ought to have for treatment of cases of heart-failure, which may come from the shock of an operation as well as from cocaine- or ether-poisoning. And if he has a fatal termination after using the best-known antidotes, his conscience will not prick him as if he actually administered poison without anything at hand for the resuscitation of the patient. This is where the danger comes in in using nostrums that are said to be harmless, but of whose composition the dentist is ignorant.

*Dr. Werner.*—The essayist spoke of a case where twenty teeth were extracted with the use of one of these preparations. Can he tell us how many subcutaneous injections were made? Would it require more for twenty-six than it would for one?

*Mr. Smith.*—Probably an intermediate number. You know, of course, how far the gum is anæsthetized by its whitened appearance, and, as it is injected on both sides of the tooth, it is hardly probable that for twenty-six teeth it would be necessary to make fifty-two injections, because one injection would probably cause numbness of the gum for some little distance beyond each tooth.

*Dr. Werner.*—Would it not seem by that that, even with "Dorsenia," where there is only two-tenths of one per cent. cocaine, it would become dangerous with twenty-six injections, or any considerable number? It seems to me the pain from the injection would be an infliction almost as bad as the extraction of the tooth.

*Mr. Smith.*—They direct the use of a very fine hypodermic needle, —the finer the needle the less pain,—and experience teaches one how to make the injection without much discomfort to the patient.

*Dr. Cooke.*—I would like to know whether any one can say whether the place in the body where the cocaine is injected would make any difference; whether, if in the region of the fifth nerve where we have to operate, it is more dangerous than it would be in other parts of the body.

*Dr. Werner.*—Of course it must. Any operation on the fifth nerve, which is in such direct sympathy with the vagus nerve,—the nerve controlling the action of the heart,—must be attended with considerable shock, or even danger.

*Dr. Brackett.*—I wish first to express my appreciation of our present Executive Committee and the evidence of the profitable work that they put before us month after month, and after that, of the specialists who have each given us the fruits and statements of positive knowledge in certain directions outside of the range of our own theorizing, our own study, or our own practice. Further than that, I wish to testify to the very great aid cocaine solutions have been to me in my practice from the time cocaine was first to be had up to the present. In saying this I do not mean that cocaine is by any means a sole reliance; but I do mean to say that it is to me one of various valuable resources. In my practice I see all classes of people, and in my humble way try to perform all kinds of operations ordinarily required of the dentist, and I should not wish to be deprived of the use of cocaine and the hypodermic syringe; I should desire not to be prevented from using nitrous oxide; I should not want to be debarred from using ether, and I should object to being prohibited from using chloroform. I find cases coming to me at short intervals in which one or another of these agents, in my judgment, educated by my experience, is better adapted than either one of the others; and, of course, I also have other cases where nothing whatever of the sort would be suitable. I think here is an illustration of the way in which humanity shows its lack of consideration, expecting everything from one agent, believing too narrowly in one doctrine or one statement, one truth or one aspect of truth. I believe that homœopathy has an element of truth in it, and so, too, have hydropathy and allopathy. I believe there is something that is true in Spiritualism; that there is also truth in the faith cure, and in the influence of mind over matter. The thing that I complain about, that I think the human mind does not generally exercise itself most profitably concerning, is that it praises a thing or condemns a thing indiscriminately, completely, and universally.

I believe that in cocaine we have an agent that has its legitimate place, and if carefully used we may expect definite, most helpful and beneficial results. In operations where we are likely to have hurts to soft tissues, such as the common application of the rubber dam where a cavity of decay has gone considerably under the gum, in the fitting of bands to roots, and wherever soft tissue in appropriate states would otherwise be hurt, cocaine is a most helpful agent; always provided, however, that it is used within the limits of safety. This is the testimony that I would give out of years of experience,—that cocaine and the hypodermic syringe have their place in the performance of dental operations, and that the dentist who does not have cocaine, or the means of making cocaine solutions, at hand is not giving himself and his patients all the comforts that they may have. That is my view of the subject; and in defending the use of cocaine I mean to be understood that this is one resource; that its use should be governed by intelligence and discrimination, and should be associated with care in the first use of the agent for a particular patient. I have found great differences in the susceptibility of patients to its influence, and until I have made myself reasonably sure of the tolerability of the drug by the patient, I seek to err on the safe side. There are a very few of my patients for whom I do not attempt to use it, and others' tolerability of the drug seems to be practically unlimited. I find that it is especially applicable in the extraction of roots, in the removal of epulis and similar tumors, and where I particularly wish to avoid haste. If I can get through very quickly the extraction of a firmly-set tooth, I may decidedly prefer nitrous oxide; but in cases where it is necessary to feel about for roots overgrown in the gum, and not requiring much strength for their extraction after they are located, nitrous oxide would be unsuited, and I find the cocaine solutions decidedly appropriate.

While I have not been so accurate about the percentage of cocaine in solutions that I employ, I have sought to have knowledge of the sum total of what I was using. Until I have proved that the patient has an especially good degree of toleration, I plan to be sure that the amount of solution which I inject for one operation represents less than the quarter of a grain of the hydrochlorate powder. I have found the pleasantness of the results very much favored by the addition of a very little carbolic acid,—a very small drop of the deliquesced carbolic acid in a drachm of solution. Credit for this suggestion belongs to our deceased member, Dr. J. W. Smith, who, within a few months after knowledge of cocaine first came to any of us, made the addition of carbolic acid, and found that the tendency to faintness, to nausea, to disturbance of the heart's action, and all the disagreeable feelings which are likely to result from the administration of cocaine, were very largely set aside by this addition of a small amount of carbolic acid. Without doubt numerous others have made the same discovery as independently as he did, but so far as I know he was the first.

*Dr. Hitchcock.*—I would like to emphasize what Dr. Allen and Dr. Brackett have just said. I have been experimenting with a weak solution,—one-fifth of one per cent.,—and have been surprised at the results. I used it to-day for the extraction of a lower molar root; the operation was painless. A severe toothache which would not yield to the usual remedies was instantly stopped by the injection of one-fiftieth of one-per-cent. solution of cocaine.

*Dr. Eames.*—After what has been stated, I cannot resist saying a word. We cannot be too emphatic in our thanks to one who has given us a practical talk on this subject. If there is one thing we should denounce with emphasis, it is preparations of a powerful drug, like cocaine, of unknown strength. The point I wish to bring up in futherance of what has been said by Dr. Hitchcock is, that we can in some cases control pain by an injection of warm water, or of cold water, into the gum. Recently a trial was made of this, and the patient said there was no pain whatever from the extraction of a tooth, the injection being made with warm water. For many years it has been the practice of surgeons to inject chloroform at the seat of the sciatic nerve to cure sciatica, using the entire length of the needle; and in some instances they have used cold water with equally good results. I speak of this in furtherance of what has been said in regard to weak solutions.

Another thought was suggested by Dr. Brackett's words as to the use of carbolic acid. It seems to me that the addition of carbolic acid localizes the effect of cocaine, and that is what we want. I have used the phenate of cocaine daily for the past two weeks, and it seems to me it should have a somewhat similar effect; perhaps some of you can testify to this. I have reason to believe that cocaine has been shamefully used. I believe that many dentists ignorantly plunge the needle into the vascular parts beyond the hard gum-tissue; then we know there is an immediate and profound effect. The place where cocaine is most effective in operations involving the lower wisdom-teeth is also the most dangerous, because the surrounding parts are very vascular. I use cocaine on an average of perhaps six times a day, and find it of great value in operations in the nose and throat. I have no doubt that Dr. Stacey could say that he has used it very often in his practice.

*Dr. Stacey.*—The cocaine which is extensively used in the eyes is ordinarily a two-per-cent. solution, although in a great many cases a four-per-cent. is used. I rather think that cocaine injected about the head is more easily absorbed, and the effects are much quicker, than when injected in any other part of the body. I have never heard of any detrimental effects to the eyes from its use. There have been cases where, in using the four-per-cent. solutions, general poisonous results have been noted, and signs of collapse have required stimulants of the heart. Aside from that, cocaine is very useful in operations about the eye.

*Mr. H. Carlton Smith.*—Of course the application which Dr. Stacey refers to is purely external, and is entirely different from an injection. I don't think there would be any serious trouble from an atropine injection, if used in such strength as Dr. Stacey has spoken of to-night.

*Dr. Eames.*—I have a formula that I have used three or four times successfully as a local anæsthetic. The constituents are, Cocaine, 2¼ grains; Tinct. belladonna, 6 drops; Carbolic acid, 1½ drops; Water, sufficient to make 1 drachm. The use of the belladonna, which is a heart-tonic, is to counteract the depressing influence of cocaine; carbolic acid is a recognized local anæsthetic, and also restricts absorption. The dose is two drops.

*Dr. Allen.*—Do you expect a specific action from each drug in that combination?

*Dr. Eames.*—I do.

*President Smith.*—I have observed in one case, where a patient had extracting done under the influence of a hypodermic injection, that though the patient did not feel the extracting of the tooth, a severe sloughing of the gum resulted. In many of these obtundents there is not only cocaine to be looked out for, but there is also some other drug which causes this severe sloughing. These preparations are also used in attempts to obtund the gum in the fitting of bands, and this severe sloughing has taken place afterwards, due to the application.

*Dr. Briggs.*—The whole thing resolves itself into the intelligent use of cocaine. We cannot get along without it very well, but its use involves a great many points, and one point is the syringe-point, which may be dirty, and that might give rise to some sloughing or troubles of that sort. One speaker said he did not see how an injection could be made without hurting the patient. It can be done without any pain if the syringe-point is entered carefully and intelligently, and there are a lot of technical points on the way to use it, from the time you fill your syringe till the operation is completed. Of course we are supposed to do all those things carefully, but the trouble is, the man who would take up with a nostrum is apt to be the man who would also take up with a dirty syringe having a blunt point, and make the injection improperly.

*Dr. Eames.*—I deaden the sensibility somewhat by the use of cotton saturated with cocaine solution applied to the gum, and that allows the introduction of the needle without discomfort. I would like to ask Mr. Smith if hydrate of chloral is not easily decomposed? This might account for sloughing in some cases.

*Mr. Smith.*—I don't know. It would depend upon what else was put into the preparation. Hydrate of chloral cannot be used internally in an alcoholic solution to any extent, because you get an alcoholate of chloral, which is far more soluble and more poisonous than is the aqueous solution. Its action upon the gum would depend very much upon the other constituents of the preparation.

*Dr. Werner.*—I would like to ask if many of these obtundent preparations contain alcohol?

*Mr. Smith.*—So far as I have tested them, they do not to any considerable extent.

*Dr. Werner.*—Are you of the opinion that "Dorsenia," for instance, contains alcohol?

*Mr. Smith.*—I think it does, but not a large percentage.

*Dr. Werner.*—A patient of mine had an aunt who had a tooth extracted with "Dorsenia," and reported very favorably about it, and this patient, having a prejudice against nitrous oxide, decided to try the same remedy. I saw him about a week after, and he said he had been complaining of a sore mouth ever since the tooth was extracted, and that satisfied him thoroughly in regard to the use of injections. The extraction did not hurt him, but the injection was quite painful, and the sloughing points were distinct after the gum-tissues around the socket of the tooth had thoroughly healed.

*Dr. Brackett.*—I express the hope that the members present do not infer that this sloughing is a regular accompaniment of the use of local anæsthetics.

*Mr. Smith.*—I would like to ask Dr. Brackett how he feels about the use of the preparations we have been discussing? So far as his ideas are concerned regarding the use of cocaine solutions of known strength, no objection can be made; but what of preparations of unknown value? How does he feel about them?

*Dr. Brackett.*—I have great satisfaction in being able to say that I have never used any one of the preparations in a single instance.

*Dr. Werner.*—The tendency is for a professional man not to use a nostrum. I think his conscience would not permit him to use anything of which he was uncertain as to its effect or its ingredients.

*Dr. Bradley.*—I have never used any of the secret preparations, though occasionally tempted to do so after receiving some of those plausible circulars. I have used cocaine more or less frequently in adjusting the rubber between the teeth or in the treatment of pyorrhœa, bathing the gums perhaps more than anything else. I have rarely injected it, and then only in a weak solution, and, as Dr. Brackett suggested, with the use of a little carbolic acid, and feel safe in doing so.

*Dr. Ainsworth.*—Is it a fact that in the face of the published analyses of preparations,—analyses,—that we really are using something the component parts of which we are in ignorance? I refer particularly to the analyses of the ten different preparations spoken of in an article by Dr. Kirk in the May (1893) *Dental Cosmos.*

*Dr. Smith.*—In certain cases, I know that the medical profession use manufactured preparations; but the formulas are published, and I suppose they consider that they are not nostrums.

*Dr. Ainsworth.*—The proprietors of these articles do not publish their formulæ; in fact, the manufacturer of one of them claims in one of his circulars, in italics, that it is actually free from cocaine, while, if the analysis is to be relied upon, it contains two-tenths of one per cent. cocaine. Now, then, suppose we accept that analysis and use that preparation, arc we using something that could be considered as an unknown preparation?

*Mr. Smith.*—Of course it is often impossible to make a complete analysis of the exact constituents. One of the preparations that I have analyzed, it is claimed, owes its efficiency to a drug that I never heard of before, and I doubt if a published analysis of such a drug could be found.

*Dr. Ainsworth.*—I have the impression that these analyses which occur in Dr. Kirk's article were made by parties whose interests were antagonistic to the manufacturers.

*Mr. Smith.*—Some substances can be detected by analysis, but other things cannot.

*Dr. Ainsworth.*—That answers my question.

*Dr. Eames.*—I hope the Academy will express its feeling with regard to secret preparations. I understand that Dr. Cooke made it as a motion that we utterly condemn secret nostrums, so-called, and to that end I should heartily second the motion.

*President Smith.*—The question before the Academy is the motion made by Dr. Cooke, that it is the sentiment of the Academy that it condemns all nostrums the analyses of which they are not familiar with.

The vote was thus carried by the Society.

*President Smith.*—We next come to "Incidents of Practice and Presentation of Specimens," and under that head I will relate to you an incident of practice and present a specimen. It is a case of bridge-work which came under my observation. A bicuspid root was implanted in the region of the upper first molar. The upper cuspid root was in place and was utilized. This implanted root, after its recovery from implantation, was banded with a gold band, and then teeth were attached from the cuspid root to a band on the implanted tooth. This was put in the mouth last May, and to-day is here for inspection. The bridge has been set in a plaster cast, just as it appeared in the mouth. You will observe the great amount of absorption that has taken place on the root of the implanted bicuspid.

*Dr. Hitchcock.*—Since hearing Dr. Whitney's paper on "Syphilis," read before the Harvard Odontological Society, I have noticed my patients' mouths more closely, and in some cases have decided to label the mouth mirror and use it only on that patient. I have also come across three or four cases of tonsillitis, in one of which the tonsils were bleeding. The mirrors used on these patients were labelled and put aside. After operating on a consumptive the same thing should be done. According to most authorities, consumption is contagious from the sputa.

I soon found that the plan of having individual mirrors for special patients, as suggested by Dr. Upham, was an expensive one. It was thus that the idea of a metallic holder for adjustable glasses suggested itself to me. In the arrangement which I have devised the glasses are detachable from a metallic holder, and cost about twice the price of the rubber dam which we use in a single operation. The holder can be sterilized by boiling.

